# A Comparative Study on Growth and Metabolism of *Eriocheir sinensis* Juveniles Under Chronically Low and High pH Stress

**DOI:** 10.3389/fphys.2020.00885

**Published:** 2020-07-21

**Authors:** Xiaodan Wang, Zhipeng Huang, Chunling Wang, Changle Qi, Zhimin Gu, Erchao Li, Jian G. Qin, Liqiao Chen

**Affiliations:** ^1^Laboratory of Aquaculture Nutrition and Environmental Health, School of Life Sciences, East China Normal University, Shanghai, China; ^2^Agriculture Ministry Key Laboratory of Healthy Freshwater Aquaculture, Key Laboratory of Freshwater Aquaculture Genetic and Breeding of Zhejiang Province, Zhejiang Institute of Freshwater Fisheries, Huzhou, China; ^3^Department of Aquaculture, College of Marine Sciences, Hainan University, Haikou, China; ^4^Department of Biological Sciences, College of Science and Engineering, Flinders University, Adelaide, SA, Australia

**Keywords:** *Eriocheir sinensis*, pH stress, antioxidant capacity, metabolic pathways, transcriptome

## Abstract

This study elucidates the effects of chronic pH stress on the growth and metabolic response of juvenile Chinese mitten crab *Eriocheir sinensis*. Crabs were exposed under normal pH (control, pH = 8.0 ± 0.20), low pH (pH = 6.5 ± 0.20), and high pH (pH = 9.5 ± 0.20) in an 8-week trial. Both low and high pH suppressed weight gain but low pH had more adverse effects. No difference was observed on survival, crude lipid, and protein. Acidic stress significantly reduced protein efficiency. The malondialdehyde (MDA) content in hepatopancreas was highest at low pH. The superoxide dismutase (SOD) activity in hepatopancreas and total hemocyte counts (THC) in the stress groups were higher than that in the control. Crabs under high pH had the highest ACP and AKP activities, but there was no significant difference between the control and low pH groups. In the transcriptome analysis, 500.0M clean reads were obtained from the control, low pH, and high pH groups, and assembled into 83,025 transcripts. Kyoto Encyclopedia of Genes and Genomes (KEGG) pathways were analyzed to obtain the significantly changed pathways involving differently expressed genes. Ten and eight pathways in metabolism were significantly changed in low pH vs control and high pH vs control groups, respectively. According to the reported functions of these pathways, most of them participated in carbohydrate metabolism. The metabolism pathway analysis indicates the increases of stress resistance, glucose metabolism, and molting activities under chronically pH stress. This study suggests that low pH has more negative impact on crab growth, and oxidative phosphorylation is the main source of energy source under low pH stress, while aerobic glycolysis supplies most energy under high pH stress.

## Introduction

Unlike seawater, freshwater has poor buffering capacity and can experience a wider range of pH fluctuation (Han et al., 2016). The level of pH in aquaculture pond fluctuates from 6.6 to 10.2 because carbon dioxide is removed by plants and algae through photosynthesis at daytime while carbon dioxide is released through respiration in the water at night (Li and Chen, 2008). The pH of surface water may also be acidic either by the natural process of organic acidity or inorganic acids from atmospheric deposition (Spyra, 2017). The pH in aquaculture ponds can increase to above 9 during the red tide formed by the rise concentration of soluble organic substances (Wang and Wang, 1995) and photosynthesis at daytime (Hayashi et al., 2012).

Generally, the exposure of freshwater organisms to an abnormal pH can lead to mortality and reduction in growth and reproduction (Kim et al., 2015). An acidic environment can reduce the pH in blood or hemolymph in aquatic animals, resulting in low oxygen carrying capacity and physiological hypoxia (Han et al., 2018a). Alkaline stress would corrode the gills of aquatic animals, leading to the reduction of ion absorption and mortality (Qian et al., 2012). Though crustaceans have the ability to adapt to pH change in a certain range, it is also evident that extreme pH especially lower pH would trigger abnormal functions in physiological, molecular, and biochemical pathways (Chen et al., 2015; Kawamura et al., 2015; He et al., 2019).

Adaptability of animals to environmental stress largely depends on the capacity of transcriptome response and gene expression (Gracey, 2007; Xu et al., 2016). RNA sequencing (RNA-Seq) has been widely applied for the transcriptome research (Wang et al., 2009). This technology provides a platform to study the stress response and adaptative mechanism for a species even without its full genome referencing database. The hepatopancreas of crustaceans is a key organ involved in digestion and detoxification, and it is also a crucial metabolic center for eliminating excess ROS and plays an important role in the immune system (Vogt, 1994; Chen et al., 2017). Therefore, in this study, hepatopancreas transcriptome analysis was taken to investigate the effects of chronic pH stress on the metabolism of crabs. The Chinese mitten crab *Eriocheir sinensis* is an important species in aquaculture and its production reached 796,622 metric tons in 2014 with a value of over 5.5 billion US dollars (Wang et al., 2019). With the development of intensive farming, pH stress has become a major concern in crab farming. Therefore, a comparative study on the metabolic response to pH stress is necessary to develop a strategy to prevent crab mortality and subsequent economic loss under a chronic low or high pH stress. Our motivation to investigate the response of crab to pH stress was due to the development of saline-alkali water aquaculture in island saline water, which has been practiced worldwide, including in Thailand, Brazil, China, Mexico, Ecuador, the United States, and Vietnam (Dinh, 2015). However, most studies on crustacean aquaculture at low salinity are mainly focusing on shrimp *Litopenaeus vannamei* (Wang et al., 2015, 2017; Chen et al., 2019) and the cultivation of crabs has not been reported (Roy et al., 2010).

## Materials and Methods

### Experimental Animals

Juvenile crabs (*E. sinensis*) were obtained from a local crab company in Shanghai, China. All crabs were acclimated in several plastic tanks (100 × 80 × 60 cm) for 2 weeks in the Biological Station of East China Normal University. Healthy crabs (450) (2.10 ± 0.20 g) were randomly assigned into 15 tanks (300 L) with five corrugated plastic pipes (12 cm long and 25 mm in diameter) and five arched tiles as the shelters to avoid attacking. Three treatments included a control (pH = 8.0 ± 0.20), low pH stress (pH = 6.5 ± 0.20), and high pH stress (pH = 9.5 ± 0.20) with five replicates each and 30 crabs in each replicate. A commercial diet with 37% crude protein and 7% crude lipid was used in this study. All the crabs were hand-fed to apparent visual satiation thrice daily at 00:00, 09:00, 17:00, and 00:00 h for 8 weeks. The volume of 1/3 to 1/2 of the tank water was exchanged and the water pH was adjusted to the target levels by adding 1 mol L^–1^ HCl or 1 mol L^–1^ NaOH stock solution. The pH was measured and adjusted every 8 h. The water quality parameters across all feeding treatments were maintained at 24 ± 1.0°C, dissolved oxygen > 7.5 mg L^–1^, and ammonia-N < 0.05 mg L^–1^.

### Sample Collection

At the end of the 8-week trial, all crabs in each tank were counted and deprived of feed for 24 h before body weight was determined. Six crabs at the stage of intermolt from each tank were randomly collected and stored at −20°C for the analysis of whole-body composition. Other six crabs from each tank were anesthetized on ice for 10 min. The 1 mL syringe with 1:1 pre-cooled anticoagulant solution (510 mmol L^–1^ NaCl, 100 mmol L^–1^ glucose, 200 mmol L^–1^ citric acid, 43.33 mmol L^–1^ citric acid, 30 mmol L^–1^ Na-citrate, 10 mmol L^–1^ EDTA⋅2Na, pH 7.3) was used to collect the hemolymph from the third pereiopod of each crab. Part of hemolymph was used for total hemocyte counts (THC), and the rest was centrifuged at 4000 × *g* for 20 min at 4°C to collect the serum. Crabs were dissected to collect the hepatopancreas, and all hepatopancreas and serum samples were stored at −80°C for further analyses. The protocols for using animals in this study were approved by the Committee on the Ethics of Animal Experiments of East China Normal University (f20190201).

### Growth Performance Evaluation and Whole-Body Composition Analysis

Survival rate (SR), weight gain (WG), and protein efficiency rate (PER) were calculated using the following formulae:

Survival rate (SR, %) = 100 × (final crab number/initial crab number);
Weight gain (WG, %) = 100 × (final weight – initial weight)/initial weight;
Protein efficiency rate (PER) = wet weight gain/dry weight of protein intake.

The body composition of crabs and the proximate composition of diets were determined according to the standard methods (AOAC) (Chem, 1992). The whole-body proximate composition was measured in four crabs from each tank and then the average per tank of the same set was used for statistical analysis. Moisture was determined by oven dry at 105°C to a constant weight. Crude protein was measured by the Kjeldahl method using KjeltecTM 8200 (Kjeltec, Foss, Sweden). Lipid was quantified by the method of Bligh and Dyer (Folch et al., 1951) using a vacuum drying oven (DZF-6050, Jinghong, Ltd., Shanghai, China). Samples were digested with nitric acid and incinerated in a muffle furnace (PCD-E3000 Serials, Peaks, Japan) at 550°C overnight for ash determination.

### Total Hemocyte Counts (THC)

Total hemocyte counts were obtained by using a hemocytometer. Each hemolymph sample was repeated three times and the mean value was recorded for statistical analysis.

### Biochemical Analysis

Superoxide dismutase (SOD) and malondialdehyde (MDA) of hepatopancreas were measured by using the iodine-starch colorimetric method with the commercial assay kits (Cat. No. A001-1, A003-1 and A005, Jiancheng, Bioengineering Institute, Nanjing, China). The acid phosphatase (ACP) and alkaline phosphatase (AKP) activity of serum were measured by the disodium phenyl phosphate hydrate method with commercial assay kits (Cat. No. A060-1 and A059-1, Jiancheng, Bioengineering Institute, Nanjing, China) according to the manufacturer’s protocols.

### RNA Extraction, cDNA Library Conduction, and Sequencing

The hepatopancreas samples were ground in liquid nitrogen, and total RNA was extracted using TRIzol^®^ Reagent in accordance with the manufacturer’s instruction (Invitrogen, United States). Extracted RNA was treated with DNase I (Takara, Japan) to remove genomic DNA. The quality and quantity of total RNA were assessed using a Nano Drop 2000 spectrophotometer (Thermo, Wilmington, DE, United States).

The RNA-seq transcriptome library was prepared following the TruSeq^TM^ RNA sample preparation kit from Illumina (San Diego, CA, United States) using 1 μg of total RNA. mRNA was isolated according to the poly A selection method using Oligo (dT) beads and then was fragmented using the fragmentation buffer.

Single-stranded cDNA was synthesized with random hexamers using RNA as a template. Double-stranded cDNA was synthesized with the effect of dNTPs, DNA polymerase I, RNase H, and buffer, and it was purified by AMPure XP beads. A single (A) was added using Klenow buffer.

Adaptor-modified fragments were selected by AMPure XP beads, and PCR amplification was performed for 15 cycles. After quantification by Qubit 2.0, the sequencing library was diluted to 1.5 ng/μL. The insert size of the library was tested by Agilent 2100, and was quantified by the Q-PCR method to guarantee the quality of the sequencing library. RNA-seq sequencing library was sequenced using Illumina HiSeq 4000. The raw sequencing data were evaluated by FAST-QC^1^ to remove low-quality reads (i.e., Q value < 20), adapter sequences, reads with ambiguous bases (“N”), and fragments < 20 bp in length. As there was no reference genome for Chinese mitten crab, the sequenced reads was spliced using Trinity first (Grabherr et al., 2011), and the hierarchical cluster analysis was used with Corset^2^ (Davidson and Oshlack, 2014).

### Gene Expression Analysis and Functional Enrichment

To identify differential expression genes between the two different treatments in two tissues, RSEM^3^ was used to quantify gene abundance. The expression level of each transcript was calculated according to the method of fragments per kilobase of transcript sequence per millions base pairs (FRKM). Differential expression analysis was conducted using DESeq2 with *p*-value ≤ 0.05. Gene Ontology (GO) analysis^4^ was performed to facilitate elucidating the biological implications of unique genes in the significant or representative profiles of the differentially expressed genes; 19 significantly changed genes were randomly chosen for validation by real-time quantitative PCR (qRT-PCR). Kyoto Encyclopedia of Genes and Genomes (KEGG) was performed for functional-enrichment analysis in the metabolic pathways at FDR ≤ 0.05. KEGG pathway analysis was carried out using KOBAS^5^.

### Statistical Analysis

All results were tested for normality and homogeneity of variance by Levene’s equal variance test. Data were presented as means ± standard error (SE). Each variable was analyzed by one-way analysis of variance (ANOVA) followed by Duncan’s multiple range test (SPSS 19.0 package; SPSS Inc., New York, NY, United States). The levels of statistical difference were set at *P* < 0.01 as extreme difference and *P* < 0.05 as significant difference.

## Results

### Growth Performance and Whole-Body Composition

**FIGURE 1:**
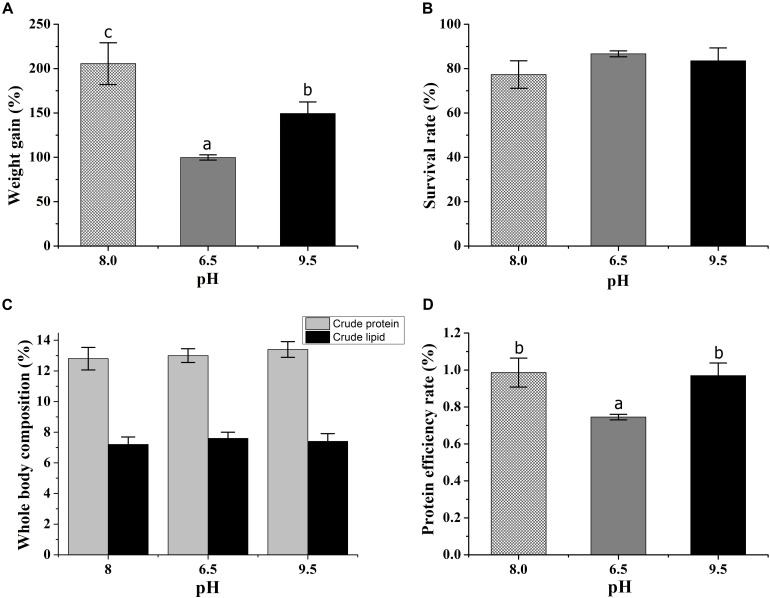
Effects of acidic and alkaline stress on the weight gain **(A)**, survival rate **(B)**, whole-body composition **(C)**, and protein efficiency rate **(D)** of *E. sinensis*. The results were presented as mean ± SE and different lowercase letters mean significant differences by Duncan’s test (*P* < 0.05).

The weight gain of the crab was significantly affected by pH stress. The crabs at pH 9.5 gained more weight than those at pH 6.5 (Figure 1A). There was no significant difference in survival rate, crude lipid, and crude protein (Figures 1B,C). The PER at low pH was lower than the control and the high pH group (Figure 1D), but there was no difference between the control and high pH group.

### Antioxidant Capacity and Immune Status

**FIGURE 2:**
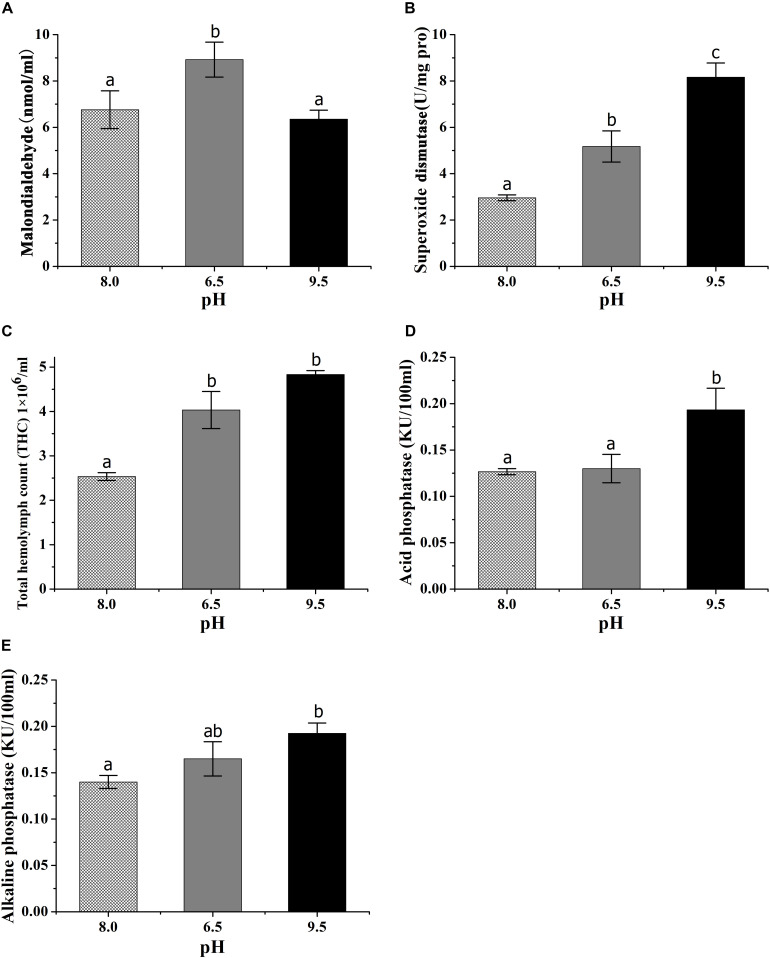
Effects of acidic and alkaline stress on the content of malondialdehyde **(A)**, the activities of superoxide dismutase **(B)**, total hemocyte counts **(C)**, acid phosphatase **(D)**, and alkaline phosphatase **(E)** of *E. sinensis*. The results were presented as mean ± SE and different lowercase letters above each column mean significant differences by Duncan’s test (*P* < 0.05).

The MDA content in hepatopancreas was highest in the pH 6.5 group and there was no significant difference between the control group and high pH group (Figure 2A). The SOD activity in hepatopancreas was higher in the high pH group than the other two groups (Figure 2B). THC in the pH stress groups were higher than those in the control group (Figure 2C). Crabs at pH 9.5 had the highest ACP and AKP activities (Figures 2D,E). There was no significant difference between the control group and low pH group in the ACP and AKP activities.

### Transcriptome Sequencing and *de novo* Assembly

A total of 175.7M, 156.2M, and 168.1M clean reads were obtained from the control, low pH, and high pH group, respectively, after the removal of low-quality reads. The mean GC (%) of these three groups was 56.80, 55.79, and 55.92%, respectively. In total, 83,025 transcripts were obtained and analyzed by *de novo* assembly (Table 1). The summary of the RNA-Seq results is shown in Table 1 and the accession number of *de novo* was PRJNA554226.

**TABLE 1:** Basic information of the transcriptome analysis.

	**Min length**	**Max length**	**Mean length**	**Percent GC**	**N50**	**Total nucleotides**	**Total numbers**
Transcripts	201	20,188	1027	46.92	1624	85,306,988	83,025
Genes	201	20,188	904	46.67	1389	58,750,515	64,995

### Analysis of Gene Expression

**FIGURE 3:**
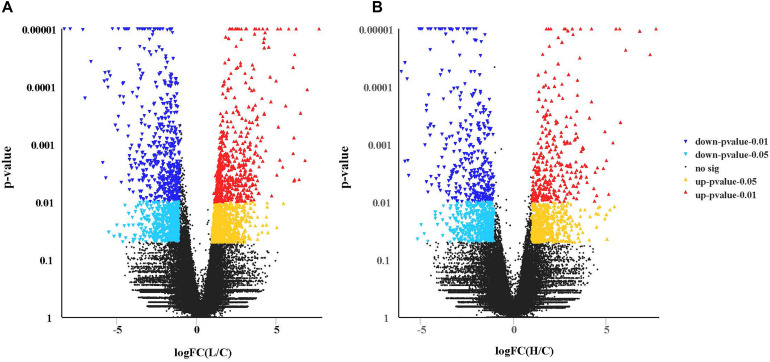
Transcriptional profiles of different expressed genes between two pair-wise comparisons in the hepatopancreas of crab revealed by Volcano plots. **(A)** L vs Control group. **(B)** H vs Control group. For volcano plots, the *X*-axis represents log (fold change), and the *Y*-axis is *p*-value. The differently expressed genes are shown as mazarine (down, *p*-value < 0.01), sky blue (down, *p*-value < 0.05), red (up, *p*-value < 0.01), and yellow (up, *p*-value < 0.05).

The mean mapping ratio of the control, low pH, and high pH groups was 82.77, 82.27, and 82.56%, respectively. The expression of 2459 genes (1344-up and 1115-down) was significantly different in the hepatopancreas between the low pH and the control groups (L pH vs Control) (*P* < 0.05, Figure 3A). The expression of 1645 genes (775-up and 870-down) was significantly different between the high pH and the control groups (Figure 3B). Log FCs from qRT-PCR were compared with the RNA-seq expression analysis results and these two results had a correlation coefficient of 0.8 demonstrating the credibility of the RNA-Seq results.

### GO and KEGG Analysis

Based on the GO analysis, the functions of the differently expressed genes could be classified into three main categories: biological process, molecular function, and cellular component. KEGG pathways were analyzed to obtain significantly changed pathways involving the differently expressed genes. Ten out of 23 significantly changed pathways were related to metabolism in the low pH vs control group, while there were 19 significantly changed pathways in the high pH vs control group and eight of them were in metabolism. According to the reported functions of these pathways, most of them participated in carbohydrate metabolism (Tables 2, 3).

**TABLE 2:** Significantly changed genes and KEGG pathways involved in metabolism response in the hepatopancreas of Chinese mitten crab under pH 6.5 vs in the control group for 8 weeks.

**Pathway term**	**Involved genes**	***P*-value**
Oxidative phosphorylation	CYC1, CYT1, petC| SDHA, SDH1| NDUFA12| COX5B| ATPeV1H| NDUFS7ATPF1D, atpH| ATPeF1B, ATP5B, ATP2| QCR2, UQCRC2| ATPeV1F, ATP6S14| UQCRFS1, RIP1, petA| QCR8, UQCRQ| SDHC, SDH3| ATPeF0B, ATP5F1, ATP4| NDUFA4| NDUFAB1| COX5A| NDUFS3| ATPeF0O, ATP5O, ATP5| ATPF0C, atpE| NDUFB2| ATPF1B, atpD| ATPeF1D, ATP5D, ATP16| ppa| NDUFB7	3.30E-06
Glycolysis/gluconeogenesis	frmA, ADH5, adhC| PDHX| LDH, ldh| E4.1.1.32, pckA, PEPCK| PGK, pgk | ENO, eno| DLAT, aceF, pdhC| PTS-Glc-EIIA, crr| GPI, pgi| ACSS, acs| PDHA, pdhA| GAPDH, gapA| DLD, lpd, pdhD| FBA, fbaA	6.07 E-04
Citrate cycle (TCA cycle)	PDHX| IDH1, IDH2, icd| E4.1.1.32, pckA, PEPCK| SDHC, SDH3| | DLAT, aceF, pdhC | MDH2| SDHA, SDH1| PDHA, pdhA| ACO, acnA| DLD, lpd, pdhD	8.39 E-04
Pyruvate metabolism	PDHX| LDH, ldh| E4.1.1.32, pckA, PEPCK| MDH2| DLAT, aceF, pdhC| ACSS, acs| PDHA, pdhA| DLD, lpd, pdhD	0.011
Starch and sucrose metabolism	UGT| UGDH, ugd| E3.2.1.28, treA, treF| E3.2.1.4| MGAM| SI| PTS-Glc-EIIA, crr| UGP2, galU, galF| GPI, pgi| TPS	0.011
Taurine and hypotaurine metabolism	CSAD| GADL1, CSADC, ADC	0.034
Glutathione metabolism	IDH1, IDH2, icd| GST, gst | E1.11.1.9| GCLC| E4.1.1.17, ODC1, speC, speF	0.042
Amino sugar and nucleotide sugar metabolism	E3.2.1.14| PTS-Nag-EIIC, nagE| HEXA_B| PTS-Glc-EIIA, crr| UGP2, galU, galF| GPI, pgi	0.045
Steroid biosynthesis	E1.14.13.72, SC4MOL, ERG25| DHCR24	0.046
Pentose and glucuronate interconversions	UGT| UGDH, ugd | DHDH| UGP2, galU, galF| DCXR	0.050

**TABLE 3:** Significantly changed genes and KEGG pathways involved in metabolism response in the hepatopancreas of Chinese mitten crab under pH 9.5 vs in the control group for 8 weeks.

**Pathway term**	**Associated genes**	***P*-value**
Starch and sucrose metabolism	MGAM| E3.2.1.1, amyA, malS| E3.2.1.28, treA, treF| SI| | PTS-Glc-EIIA, crr| GPI, pgi| TPS	1.75 E-04
Glycolysis/gluconeogenesis	ENO, eno| E4.1.1.32, pckA, PEPCK| GAPDH, gapA| ADPGK| FBA, fbaA| LDH, ldh| PTS-Glc-EIIA, crr| PGK, pgk| GPI, pgi| GAPDH, gapA| gpmI	8.07 E-04
One carbon pool by folate	FTCD| MTHFS| metF, MTHFR	0.010
Amino sugar and nucleotide sugar metabolism	nagA, AMDHD2| PTS-Nag-EIIC, nagE| PTS-Glc-EIIA, crr| GPI, pgi| manA, MPI	0.010
Linoleic acid metabolism	PLA2G, SPLA2| CYP2J| CYP3A	0.015
Galactose metabolism	MGAM| SI	0.025
Glycosaminoglycan degradation	NAGLU| SGSH	0.043
Taurine and hypotaurine metabolism	CSAD	0.048

## Discussion

A pH value between 6.5 and 9.0 is satisfactory for most freshwater animals in long-term of farming practice (Alabaster and Lloyd, 1980). Moreover, a high pH value of 9.5 is quite common in ponds for crab farming (Kong et al., 2012). In this study, there was no significant difference in growth performance parameters between any pH groups after 8 weeks, indicating the Chinese mitten crab can survive in pH 6.5–9.5. But there was also a study showing that 40% healthy crabs (about 50 g) died after exposure to pH 9.5 for 2 weeks (Pan et al., 2017). That is because smaller crabs have greater tolerance to environmental stress than bigger ones (Tood and Dehnel, 1960). Weight gain was reduced by water pH stress, and the acidic pH had more negative effect, which is consistent with the result of pH effect on protein utilization. The negative effect of pH stress may be mediated by the disruption of metabolic regulation, extra energy expenditure, and reduction of nutrient absorption (Leal et al., 2011), which may explain why weight gains in the low pH and high pH groups are low in the current study. The other possible reason may be that acidification may alter the dynamics of muscle water content, which would lead to the changes of weight gain.

Environmental stress can trigger the over production of reactive oxygen species (ROS) and result in a severe damage to cells (Du et al., 2013). MDA is one of the most known products of lipid peroxidation and is a well-established marker of oxidative stress, which can be induced by a wide range of environmental stress (Wang et al., 2004). Therefore, the increase of MDA is usually associated with various environmental stress and the pathological state of animals (Zhang Y. et al., 2015). Aquatic crustaceans lack an adaptive immune response and mostly rely on innate immune responses, among which the antioxidant defense system is important to reduce ROS damage (Muta and Iwanaga, 1996; Zhang W. et al., 2015). SOD is a key antioxidant enzyme directly participating in the removal of ROS (Kobayashi et al., 2019). ACP and AKP are highly conserved enzymes and play an important role in the non-specific immunity of crustaceans (Yang et al., 2007; Gisbert et al., 2018). Thus, higher ACP and AKP activities have a positive effect on the defense against external microbial invasion (Wu et al., 2019). Total hemocyte numbers of crustaceans can vary greatly after exposure to environmental stress and infections by bacteria, fungi, and viruses (Truscott and White, 1990; Vargas et al., 1997; Iwanaga and Lee, 2005; Vazquez et al., 2009). Any increase in THC could lead to an increase of immune defense ability (Min et al., 2015). The results of the present study reveal that all three immune parameters of the crabs were significantly affected after exposure to alkaline acid stress. Also, an increase of OH^–^ concentration seems to have more stimulative effects on immune function of the crabs than those at a high H^+^ concentration, which may be the reason why the MDA content in the high pH group was lower than that in the low pH group.

When suffering in environmental stress, crustaceans require additional energy to maintain homeostasis (Wang et al., 2016). Carbohydrates are often included in artificial diets to serve as an energy source, and they can also meet the high energy demand of aquatic animals under stress (Tseng and Hwang, 2008; Wang et al., 2017). Mitochondrial oxidative phosphorylation provides over 90% of the energy produced by aerobic organisms; therefore, the regulation of oxidative phosphorylation is a major issue for coping with the environment changes to meet more energy need (Bermejo-Nogales et al., 2015). However, animals would use glycolysis to produce energy in the absence of adequate oxygen. In the presence of adequate oxygen levels, the intracellular pH might be a factor that determines which way to obtain energy. For instance, oxidative phosphorylation could be driven in an acidic condition and aerobic glycolysis is driven in an alkaline condition (Khalifat et al., 2008; Calderon-Montano et al., 2011). Similar to these findings, the significant changes in oxidative phosphorylation, TCA cycle, and pyruvate metabolism pathways at low pH may be driven by the acidic medium, and the glycolysis/gluconeogenesis pathways can be driven by high pH. However, the primary response in aquatic animals is usually accompanied with high levels of plasma glucose and anaerobic glycolysis (Arends et al., 1999; Acerete et al., 2004; Fazio et al., 2015). This may explain why both oxidative phosphorylation and glycolysis/glycogenesis pathways were upregulated in the low pH group. Due to the enhanced glucose activities, the digestion of carbohydrates was increased in terms of the upregulated starch and sucrose metabolism pathways in both acidic and alkaline groups.

Except for conventional carbohydrates, the presence of non-glycogenic carbohydrates also contributes to the concentration of total carbohydrates in the hepatopancreas of most crustaceans (Chang and O’Connor, 1983). The amino sugar glucosamine (GlcN) and *N*-acetylglucosamine (GlcNAc) are prevalent in the biosphere. For instance, amino sugars are the major component of the exoskeleton of crustaceans (Zeng et al., 2016). The changes in the amino sugar pathways in the current study indicate that acidic and alkaline stress might affect the molt activity of Chinese mitten crab. Similarly, some other studies also found that environmental stress would change molting frequency in ammonia-exposed *Penaeus monodon*, saponin-exposed *Penaeus japonicus*, copper sulfate- exposed *P. monodon*, nitrite-exposed *P. monodon*, and acid-exposed *Macrobrachium rosenbergii* (Chen and Chen, 1992, 2003; Chen and Lin, 1992). In the current study, the finding is further verified by the changes of pentose and glucuronate interconversions, galactose metabolism pathways, and steroid biosynthesis pathways in low pH and high pH groups.

Glycosaminoglycans are heteropolysaccharides composed by a repeat disaccharide unit in which one of the two monosaccharides always contains an amino sugar (*N*-acetylgalactosamine or GlcNAc) (Antonio et al., 2013). A previous study reported that the crayfish *Orconectes virilis* might depend on the pentose pathway during intermolt and glycolysis during premolt in carbohydrate metabolism (Mcwhinnie, 1962). The glycolytic, pentose phosphate, and glucuronate pathways are operated as catabolic pathways for glucose utilization in intermolt crayfish, *Pacifastacus leniusculus* (Puyear et al., 1965). Galactose also appears frequently during the premolt of period to satisfy the carbohydrate need of molting (Vernberg and Vernberg, 1972).

As a member of gonad stimulatory hormones, steroid plays a pivotal role in molting and reproduction in crustaceans (Subramoniam, 2017). Steroid hormones also have antioxidant and free radical scavenging activities and the physiological metabolisms involved in steroid in the hepatopancreas of crustacean would promote SOD activity (Cai et al., 2002; Wu et al., 2016). The SOD activity of the crab in low pH group was enhanced in the current study. Enzymes such as SOD, glutathione peroxidases (GPx), and some non-enzymatic defense such as glutathione (GSH), tocopherols, taurine, and urate constitute the first line of defense to inactive ROS and scavenge free radicals (Sharma et al., 2004; Wang et al., 2019). The thiol group in the cysteine moiety of GSH is a reducing agent and can be reversibly oxidized and reduced (Meister and Anderson, 1983). However, GSH is an acidic molecule characterized by a γ-linked amino acid, and the cysteine residue can reduce its stability in an alkaline environment (Anik et al., 2016; Giustarini et al., 2016). That is why the GSH metabolism pathways were significantly changed in the low pH group but not in high pH group. Taurine, hypotaurine, and their metabolic precursors (cysteamine, cystic acid, and cysteinesulfinic acid) may protect the organisms against a variety of oxidants (Konukoglu et al., 1999). As antioxidants could protect the crustaceans from oxidative stress, the taurine and hypotaurine metabolism pathways were significantly changed in both low and high pH groups.

Some crustaceans could also convert the linolenic acid to the highly polyunsaturated fatty acids such as eicosapentaenoic acid in the similar way to what has been reported in fish (Kabeya et al., 2018). Long chain polyunsaturated fatty acids (LC-PUFA, involved in eicosapentaenoic acid) have an important role in physiological processes such as immune and stress responses (Tocher, 2010; Norambuena et al., 2015). The changes of the linoleic acid metabolism in the high pH group indicate that alkaline stress may stimulate *de novo* synthesis of EPA, and improve the ability of stress resistance in crab.

In general, the crab growth was suppressed by both acidic and alkaline stress, especially at pH 6.5. The increase of OH^–^ concentration seems to have a more stimulative effect on antioxidant ability and immunity and stronger ability of stress resistance than the crabs under acidic stress. Environmental stress would increase the metabolic activity of animals to compensate the change and maintain homeostasis (Koehn and Bayne, 1989). In the current study, data of transcriptome reveal that the main metabolic changes were the pathways related to glucose metabolism as carbohydrates can rapidly provide energy to meet the high energy demand of aquatic animals in a stress condition. Oxidative phosphorylation might be the main source of energy under acidic stress, while the aerobic glycolysis supplies most energy during alkaline stress. The pathway analysis indicates that pH stress may affect the molting process, but this claim needs a longer term field study to confirm.

## Data Availability Statement

The datasets generated for this study can be found in the PRJNA554226.

## Ethics Statement

The animal study was reviewed and approved by the Committee on the Ethics of Animal Experiments of East China Normal University.

## Author Contributions

XW, ZH, EL, and LC designed the research. XW, ZH, CW, and CQ conducted the research and contributed to the data acquisition and analysis. XW and LC contributed to the draft and the final writing of the manuscript. ZG and JQ revised the manuscript. All authors agreed to be accountable for all aspects of the work and approved the final manuscript.

## Conflict of Interest

The authors declare that the research was conducted in the absence of any commercial or financial relationships that could be construed as a potential conflict of interest.

## Footnotes

1 http://www.bioinformatics.babraham.ac.uk/projects/fastqc/

2 https://code.google.com/p/corsetproject/

3 http://deweylab.biostat.wisc.edu/rsem/

4 http://www.geneontology.org/

5 http://kobas.cbi.pku.edu.cn/home.do
